# A Novel Adhesion Index for Verifying the Extent of Adhesion for the Extensor Digitorum Communis in Patients with Metacarpal Fractures

**DOI:** 10.1038/srep31102

**Published:** 2016-08-05

**Authors:** Ting-Yu Lai, Hsiao-I Chen, Cho-Chiang Shih, Li-Chieh Kuo, Hsiu-Yun Hsu, Chih-Chung Huang

**Affiliations:** 1 Department of Biomedical Engineering, National Cheng Kung University, Tainan, Taiwan; 2 Department of Occupational Therapy, National Cheng Kung University, Tainan, Taiwan; 3 Department of Physical Medicine and Rehabilitation, National Cheng Kung University Hospital, College of Medicine, National Cheng Kung University, Tainan, Taiwan

## Abstract

This study aims to determine if the relative displacement between the extensor digitorum communis (EDC) tendon and its surrounding tissues can be used as an adhesion index (AI) for assessing adhesion in metacarpal fractures by comparing two clinical measures, namely single-digit-force and extensor lag (i.e., the difference between passive extension and full active extension). The Fisher–Tippett block-matching method and a Kalman-filter algorithm were used to determine the relative displacements in 39 healthy subjects and 8 patients with metacarpal fractures. A goniometer was used to measure the extensor lag, and a force sensor was used to measure the single-digit-force. Measurements were obtained twice for each patient to evaluate the performance of the AI in assessing the progress of rehabilitation. The Pearson correlation coefficient was calculated to quantify the various correlations between the AI, extensor lag, and single-digit-force. The results showed strong correlations between the AI and the extensor lag, the AI and the single-digit-force, and the extensor lag and the single-digit-force (*r* = 0.718, −0.849, and −0.741; *P* = 0.002, *P* < 0.001, and *P* = 0.001, respectively). The AI in the patients gradually decreased after continuous rehabilitation, but remained higher than that of healthy participants.

Injuries to the hand, wrist, and fingers account for more than one million emergency department visits by workers annually in the United States^1^. These injuries often involve damage to multiple tissues because of the affected area being surrounded by soft tissues. Metacarpal fractures constitute 30% to 36% of all hand fractures^2^. Adhesion is a complication that may follow fractures and is defined as the formation of a pseudoinsertion at the tethered site on a tendon that obstructs the distal and proximal gliding of the tendon^3^. Adhesion can lead to suboptimal rehabilitation outcomes. A potential risk after metacarpal fracture is tendon adherence between the tendon and the fracture callus or surrounding scar tissue^2^.

Many methods have been reported for the quantitative assessment of the extent of tendon adhesion, including measuring the work output of the digital flexion at maximal digital flexion^4^, gliding excursions by fixed loading^5^, adhesion breaking strength^6^, flexor digitorum profundus (FDP) tendon excursion with free motion of the chicken toe^7^,^8^,^9^ and simultaneous measurement of the flexion angle and gliding force with unrestricted movement of the chicken toe^10^. However, most of these studies entailed invasive experiments and animal testing and did not involve human patients; most importantly, they performed only gross evaluations of the extent of tendon adhesion. Although one study successfully investigated the dynamic nature of tissue strains in adhesion at a local level in rabbits^11^, the method employed was extremely invasive and thus difficult to apply in daily clinical settings for routine rehabilitation.

Currently, no method is available for noninvasively evaluating the gross extent of adhesion while identifying the location on the tendon suspected of the formation of adhesion. A method that provides information about the extent of adhesion at both the macro and micro levels can assist clinicians in making better-informed decisions in rehabilitation processes and thereby improve the quality of health care. However, ultrasound imaging is an established method for the accurate diagnosis of tendon pathology^12^,^13^,^14^,^15^. A retrospective review of the radiographic, clinical, and surgical records of patients referred for finger sonography over a 2-year period revealed that ultrasound imaging correctly identified tendon rupture or adhesive scarring in 27 of 28 digits with 1 false-positive case (sensitivity, 100%; specificity, 93%; positive-predictive value, 93%; negative-predictive value, 100%; accuracy, 96%)^16^.

A recent study using ultrasound found that the total displacement of the surrounding tissue of the FDP tendon constituted 22% to 53% of the total displacement of the FDP tendon in 11 healthy human control subjects^17^, agreeing with results obtained in previous studies^18^,^19^,^20^,^21^. However, this study only estimated displacement at one tendon location instead of all possible locations. In addition, no patients were included in that study; hence, whether the relative displacement can be used to evaluate the extent of adhesion and identify locations suspected of the formation of adhesion remains uncertain.

The present study aims to test the hypothesis that the relative displacement between the extensor digitorum communis (EDC) tendon and its surrounding tissues can be used as an adhesion index (AI) for determining the extent of adhesion in patients with metacarpal fractures and identifying the locations of suspected adhesions. Emphasis is placed on verifying whether this index is correlated with extensor lag and single-digit-force, which are two clinical measures currently used for evaluating the gross extent of such adhesion^2^.

## Materials and Methods

### Study Groups

The Institutional Medical Ethics Committee of National Cheng Kung University approved this prospective cross-sectional study, and written informed consent was obtained from each participant. The study was conducted between December 25, 2014 and November 20, 2015. The experimental methods were carried out in accordance with approved guidelines and regulations^22^,^23^.

The following inclusion criteria were applied when recruiting patients suffering from metacarpal fractures: (a) aged 20–65 years and (b) able to fully understand and cooperate in the experiment. The exclusion criteria were (a) a history of related hand injuries or surgeries, (b) a history of rheumatoid arthritis, diabetes mellitus, or osteoporosis, or (c) the presence of central nerve injuries. The presence of central nerve injuries was adopted as an exclusion criteria because patients with central nerve injuries such as cerebral vascular accident and traumatic brain injury tend to have symptoms of hemiplegia; this would have been disadvantageous to the present study because measurements of the single-digit-force of the injured finger were normalized by comparing them to those of the contralateral normal finger. To evaluate the performance of the proposed AI for assessing the change in the extent of adhesion, ultrasound image sequences and measurements of the single-digit-force and extensor lag were acquired from each patient twice (hereinafter referred to as the first and second measurements) with an interval of 2 weeks (during which continuous rehabilitation was applied). In total, 8 patients were included, which entailed 1 index finger, 4 ring fingers, and 3 little fingers being assessed.

The inclusion and exclusion criteria for the healthy participants were the same as those for the patients, except that a requirement for a normal range of finger motion was added to the inclusion criteria for the healthy subjects. A total of 39 healthy participants were included and ultrasound image sequences were obtained from each of them. The distribution of their digits was matched according to the distribution in the patients. Thus, 2 index fingers, 31 ring fingers, and 6 little fingers were assessed. The healthy participants were tested only once (the patients were tested twice to evaluate the performance of the proposed AI in assessing the change in the extent of adhesion). However, 10 randomly chosen healthy participants were tested twice to evaluate the reliability of the proposed AI by calculating the intra-class correlation coefficient. Tests of extensor lag and single-digit-force were not applied to the healthy subjects (these were included in patient experiments to compare their ability to evaluate the extent of adhesion to the AIs).

The age, sex, dominant hand, and digit involved in the experiment of each patient and healthy individual were recorded and a chi-squared test was performed to ensure the homogeneity of the two groups. The duration of injury of each patient was also recorded for descriptive statistics.

General principles for the postoperative care of metacarpal fractures

The rehabilitation guidelines complied with the general principles prescribed in textbooks^22^,^23^. All of the protocols were based on these general principles, with few variations for different injury types. The general principles for the postoperative care of metacarpal fractures are described as follows:

4 weeks

- Protection with splint, Controlled stabilized joint motion (passive ROM), Scar management

6 weeks

- Gentle progressive mobilization (active and passive ROM), Stretch and tendon gliding exercise, Scar management

8 weeks

- Strengthening, Activities of daily lives training, Resistance exercise

12 weeks

- Strengthening, Dexterity training

### Morphology of the EDC tendon

The EDC tendon is usually considered as one muscle with four tendons because of the incomplete separation between the tendons’ muscle bellies within the forearm^24^. The EDC tendon originates from the lateral epicondyle of the humerus, passing through the extensor retinaculum at the dorsum of the hand, then dividing into four separate tendons. The tendons then split into one central and two lateral slips attaching to the dorsal base of the middle and distal phalanxes of the index, middle, ring, and little fingers. The primary function of the EDC tendon is the extension of the digits, and it also abducts the digits as it extends.

### Motion Protocols

**Figure 1:**
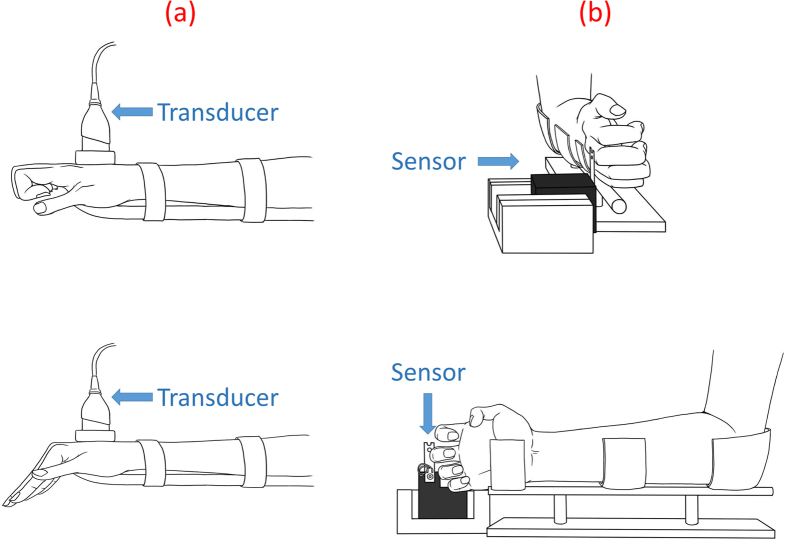
Schematic of a patient’s hand adopting an intrinsic-minus position for extension and intrinsic-plus position for flexion (**a**), and of a single-digit-force being measured (**b**).

In the intrinsic plus position, which is also known as the safe position, the metacarpophalangeal joints are flexed at 70–90° and the interphalangeal joints are fully extended. While in the intrinsic minus position (claw posture), the metacarpophalangeal joints are extended and the interphalangeal joints are flexed^3^. EDC tendon motion was measured by first asking the patient to place the hand in the intrinsic plus position to produce flexion of the metacarpophalangeal joint (MCPJ) and then the intrinsic-minus position to produce extension of the MCPJ. The intrinsic minus position produces EDC tendon glide over the metacarpal bone. Prior to making the recordings, the required flexion/extension speed of the movement (5 mm/s) was practiced with the patients following verbal instructions from the examiner. The EDC tendon in the hand was then located by placing the ultrasound transducer on the dorsal side of the patient’s hand proximal to the wrist, as shown in Fig. 1(a). Before the images were obtained, the patient was asked to initially place the hand in the intrinsic-plus position. The patient was then asked to adopt the intrinsic-minus position to promote EDC tendon glide over the metacarpal bone, and requested to hold this position for 3 s before returning to the original intrinsic-plus position. The examinations were performed by a single ultrasound expert (C.C.H., who has 10 years of experience in ultrasound imaging). The motion of the EDC tendon in an ultrasound image is prone to interference from the motion of its surrounding muscles because of the specific hand motion required to promote EDC tendon glide over the metacarpal bone^3^; this problem was overcome when making the recordings by employing an experimental procedure utilizing customized splints. Because the primary function of the EDC tendon is digit extension, a customized short arm splint was used to restrict the movement of the wrist joint and forearm to produce finger movement separately. Thus, the influences from adjacent joints were averted.

### Ultrasound Recordings and Imaging Analyses

All of the ultrasound images were acquired at 18 FPS using a Terason T3000 device (Teratech Corporation, Massachusetts, USA) with a multifrequency transducer (5–12 MHz, 12L5). All of the obtained image sequences were exported as uncompressed AVI files and imported into MATLAB software (version R2013a, The MathWorks, Massachusetts, USA) for additional analyses. Each image sequence typically contained 200 images.

**Figure 2:**
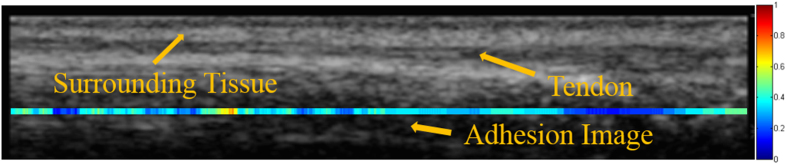
Ultrasound image of the EDC tendon and its surrounding tissue from a healthy individual. The surrounding tissue can be identified by its hyperechogenic structure while the tendon can be recognized by its less echogenic characteristics, fiber-like structure, and larger movement compared to other tissues in the region. The adhesion image indicates the locations of the tendon where the occurrence of adhesion is suspected; that is, where the relative displacement ratio is >0.6. A smaller ratio indicates a better gliding ability (relative displacement ratio of <0.6), and so the adhesion image for a healthy individual would generally appear green or blue.

The EDC tendon and its surrounding tissue were identified by a single clinician (L.C.K., who has 18 years of experience as a hand therapist). An example of how the EDC tendon and its surrounding tissue were identified in a B-mode ultrasound image obtained from a healthy individual is shown in Fig. 2. Displacement estimates were obtained using the Fisher–Tippett block-matching method^25^, with a Kalman-filter algorithm being subsequently applied to reduce tracking errors^26^. The parameter settings for the Fisher–Tippett block matching method are shown in Table 1. The parameter settings for the Kalman filter were the same as those applied in Lai^26^ except that the additive white noise of *R* and the covariance matrix of the white sequence of *Q* were set at 1 and 0.005, respectively.

**Figure 3:**
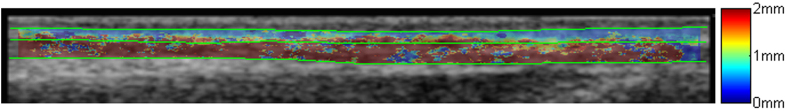
Displacement distribution of the locations along the tendon and its surrounding tissue in a healthy individual. The displacement is larger at most locations within the tendon than in the surrounding tissue (relative displacement ratio of >0.6).

The displacement estimate of each reference template represented the displacement of the center pixel of that reference template. By maximal overlapping of the reference windows, the displacement of all pixels along the tendon and its surrounding tissue could be obtained (Fig. 3).

To calculate the AI, all optimized displacement estimates in the *axial* direction in each group (the tendon and the surrounding tissue) were first averaged to obtain the displacement of the specific *lateral* location (hereinafter referred to as the *x*-location) in each group. The adhesion image *A*(*x*) was then obtained by calculating the ratio of the total displacement of the tendon to its surrounding tissue at locations along the *x* direction:


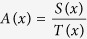


where *S*(*x*) and *T*(*x*) are the averaged total displacements of the surrounding tissue and the tendon, respectively, at location *x* along the tendon. Typically, approximately 600 *A*(*x*) locations were measured along the tendon. An example adhesion image obtained from a healthy subject is shown in Fig. 2.

On the basis that this ratio ranges were reported from 0.22 to 0.53 in healthy human subjects^17^ and the measurement taken in 39 healthy individuals in the present study (mean, 0.49 [IQR, 0.46–0.51]) corroborated this, any ratio exceeding 0.6 at any location along the tendon was assumed to be indicative of the occurrence of adhesion. The AI of the tendon was thus obtained by calculating the percentage of *A*(*x*) locations that were larger than 0.6. The methodology for finding the percentage of locations that were above this threshold was selected because of its accuracy in determining the extent of the total coverage area on the tendon that exhibited adherence.

### Extensor Lag

Extensor lag is a common sequela of metacarpal fractures that occurs when the active extension is smaller than the full passive extension. Because adhesion may decrease the gliding amplitude of the EDC tendon, it may reduce the active extension range of motion of the digits. A previous study found that metacarpal fractures frequently lead to extensor tendon dysfunction through adhesion^2^. Hence, extensor lag has been used in clinical applications to evaluate the extent of adhesions on the EDC tendon^2^. The extensor lag is defined as the difference between passive extension and full active extension when the passive extension of the finger exceeds the active motion^27^; this was measured at the MCPJ and the interphalangeal joints (IPJ) using a goniometer (an instrument used for measuring the angles of human joints in clinics) to evaluate the functionality of the EDC tendon after rehabilitation.

### Single-Digit-Force

The function of a tendon is the transfer of force generated by a muscle to a connected bone so as to induce movement. Therefore, the magnitude of the single-digit-force was used as an outcome measure for evaluating the functionality of the tendon. A full-bridge thin-beam force sensor (TBS-40, Transducer Techniques, California, USA) was used to calculate the single-digit-force by measuring the voltage change induced by the load. The single-digit-force was measured when the patient used their proximal phalangeal to exert a maximal voluntary force on the sensor under the hook position (Fig. 1(b)); this action is defined as having the metacarpophalangeal joint extended at 0° (neutral position) and interphalangeal joints flexed maximally^28^. Three measurements of the single-digit-force exerted by each patient were averaged to reduce errors. However, according to previous studies, hand strength varies substantially among people; our findings confirm this. Although no consensus has been established on whether the dominant hand is 10% stronger than the nondominant hand, variation in measurements between the dominant and the nondominant hand in most studies exhibit a smaller variation compared to the variation in hand strength among people^29^,^30^. In addition, many studies have utilized the methodology of comparing the injured site to the contralateral healthy site when assessing the rehabilitation progress^31^,^32^. Therefore, each of these averaged measurements was normalized by calculating the ratio relative to the value measured for the contralateral healthy finger.

### Statistical Analysis

Statistical analysis was performed using SPSS software (version 17.0, IBM, Illinois, USA). Descriptive statistics were used to summarize demographic and duration-of-injury information. Because a preliminary Shapiro-Wilk test demonstrated that the variables conformed to a normal distribution, Pearson’s correlation coefficients were calculated to quantify the correlations among the AI, extensor lag, and single-digit-force. A correlation was considered relevant when *r* > 0.7 or *r* < −0.7. The intraclass correlation coefficient was calculated to determine the test–retest reliability of the proposed AI. A value above 0.75 was considered to indicate an acceptable reliability. Because of the low sample size, the Mann-Whitney test and Wilcoxon signed-rank tests were used to compare the AIs of healthy subjects to those of patients as well as the respective AIs of separate pairs of measurements. Two-tailed tests were used for all statistical analyses (α = 0.05). In addition, in clinical observations, the limitation of the joint motion and restrained force exertion of the hand have usually been described as having an association with tendon gliding conditions. However, the relationship among these independent factors with respect to tendon gliding and adhesion has not been quantitatively explored using different measurements such as length, goniometry, and force. This study applied the correlation test, which is a straightforward method for examining the relationship among the three variables (AI, extensor lag, and single-digit-force) and investigating the ability of the proposed AI to assess the recovery status of the patients. In addition, data obtained in the first and second measurements were combined and correlated to explore the relationship among the variables. In other words, for demonstrating the recovery status of the patient at different time points each measurement of AI, extensor lag, and single-digit-force in the present study was considered independent.

## Results

### Comparison of AI and Current Clinical Evaluations

**Figure 4:**
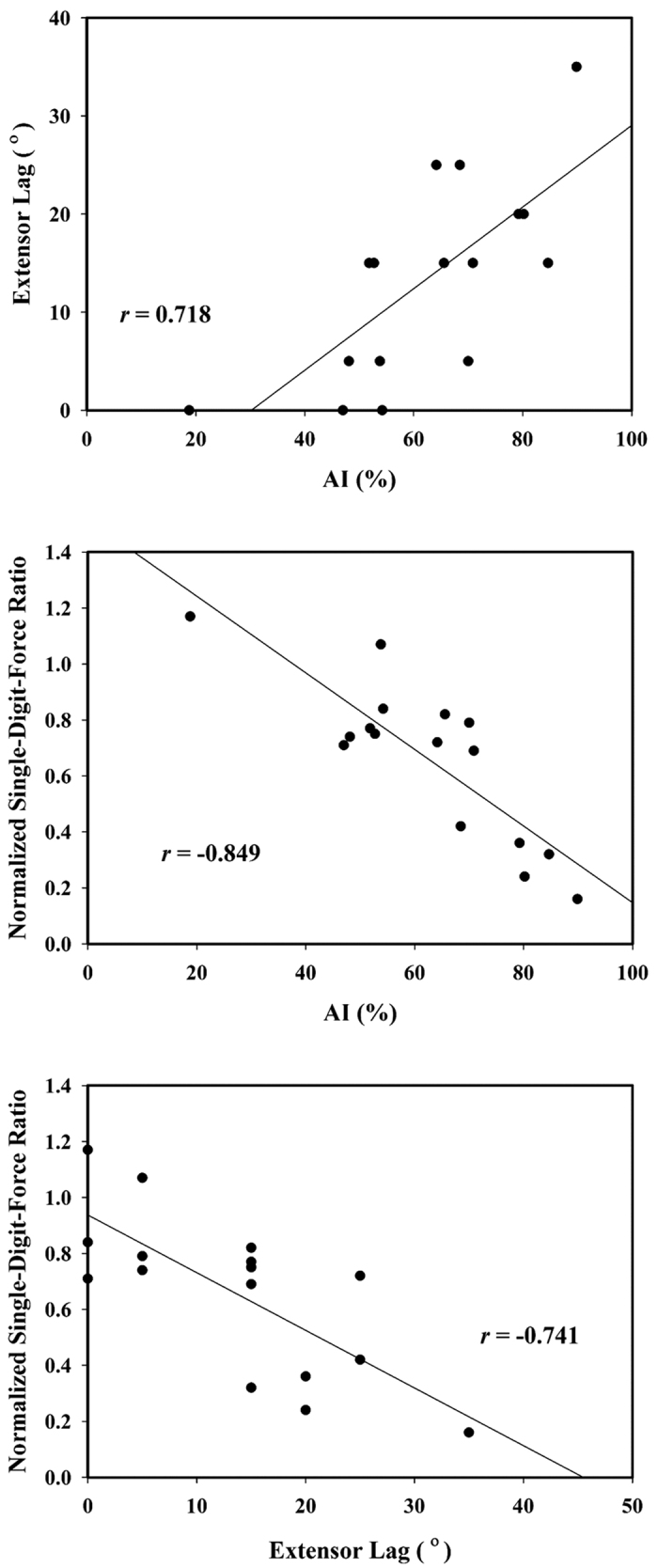
Scatter plots of the AI vs the normalized single-digit-force ratio, the AI vs the extensor lag, and the extensor lag vs the normalized single-digit-force ratio.

The results obtained by analyzing 16 ultrasound recordings as well as the measurements of the normalized single-digit-force ratio and the extensor lag are listed in Table 2; none of the ultrasound recordings were excluded. The mean AI, extensor lag, normalized single-digit-force ratio, and patient age were 62.47% (IQR, 52.50–72.96%) 13° (IQR, 5–20°) 0.66 (IQR, 0.41–0.80) and 43 years (IQR, 28–57 years) respectively. The results in Fig. 4 illustrate the strong correlations between the AI and the extensor lag, the AI and the single-digit force, and the extensor lag and the single-digit force (*r* = 0.718, −0.849, and −0.741; *P* = 0.002, *P* < 0.001, and *P* = 0.001, respectively).

### Comparison of Patients and Healthy Individuals

**Figure 5:**
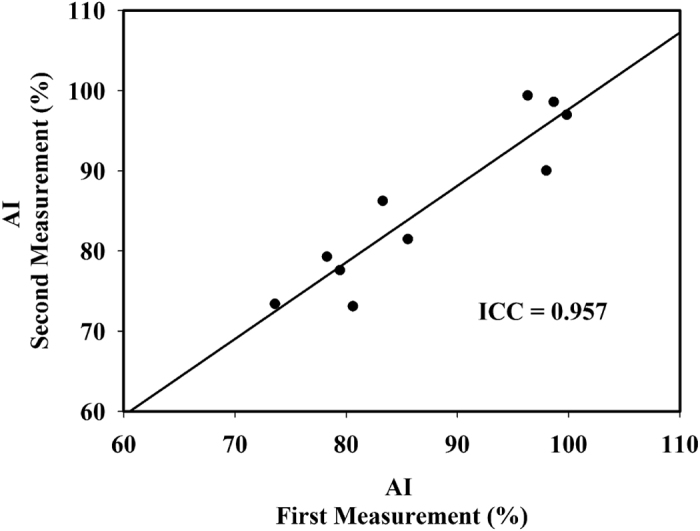
The plot of the intraclass correlation coefficient between the first measurement and the second measurement of the 10 randomly chosen healthy individuals.

**Figure 6:**
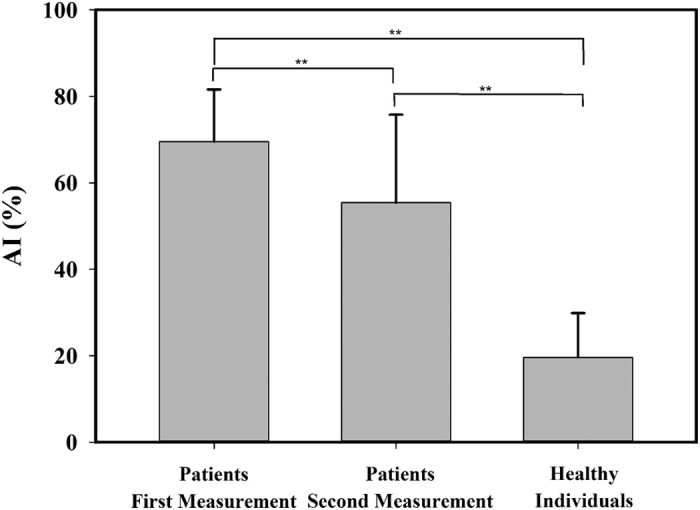
Bar chart of the AI for the first and second measurements of the patients and for the healthy individuals. (**P < 0.01).

**Figure 7:**
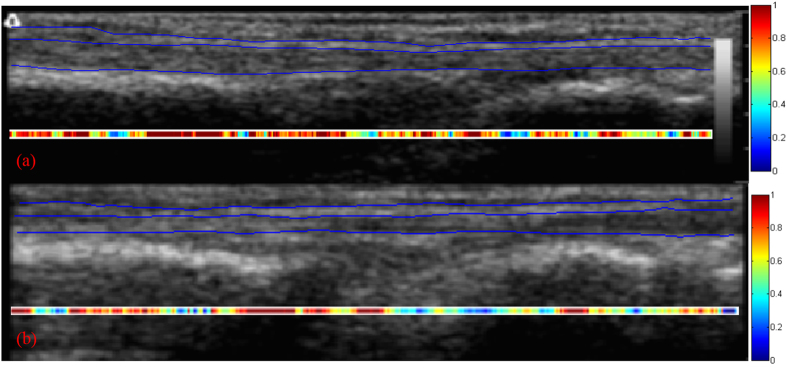
Ultrasound image of the EDC tendon and its surrounding tissue from the first (**a**) and second (**b**) measurements of a patient. The marked difference between the two adhesion images illustrates the improvement after rehabilitation.

The results obtained by analyzing 39 ultrasound recordings from the 39 healthy individuals are listed in Table 2; none of the ultrasound recordings were excluded. The intraclass correlation coefficient was 0.957, indicating substantial reliability between the first measurement and the second measurement of the healthy individuals (Fig. 5). The mean AI and age of the healthy subjects were 19.6% (IQR, 12.52–26.54%) and 36 years (IQR, 24–47 years), respectively. Age, sex, dominant hand, and involved digit did not differ significantly between the patients and healthy subjects (*P* = 0.569, 0.269, 0.196, and 0.219, respectively) (Table 3). Figure 6 is a bar chart of the AI for the first and second measurements of the patients and for healthy subjects. The AI was mainly smaller in the healthy subjects (mean, 19.6% [IQR, 12.52–26.54%]) than in the patients (62.47% (IQR, 52.50–72.96%)]). Significant differences were identified between the first and second measurements of the patients, the first measurements of the patients and the healthy individuals, and the second measurements of the patients and the healthy individuals (*P* = 0.017, *P* < 0.001, and *P* < 0.001, respectively). Typically, the AI was smaller in the second measurement of the patients (mean, 55.41% [IQR, 50.62–60.72%]) than in the first measurement (mean, 69.54% [IQR, 65.21–72.96%]). An example adhesion image obtained from a patient before (first measurement) and after (second measurement) rehabilitation is shown in Fig. 7.

## Discussion

This study found that the AI was strongly correlated with two other clinical measures, the single-digit-force and the extensor lag, and thereby confirms the hypothesis that the proposed AI can be used to evaluate the extent of adhesion in patients with metacarpal fractures. Specifically, the extent of adhesion indicates the extent of the coverage area on a tendon exhibiting adherence. However, the correlation between the AI and the extensor lag was slightly weaker than that between the AI and the single-digit-force. This could be caused by the extensor lag being a less sensitive clinical measure than the single-digit-force is. From an anatomical perspective, tendons connect bone and muscle with parallel fibers and the biomaterial to transmit tensile forces from muscle to bone and support digit movement. The digit force is not fully transmitted after an adhesion has formed. The obstruction of the force transmission by adhesions has been confirmed by the biomechanical model proposed in previous research^33^. However, when the robustness of the adhesion has decreased, the gliding ability of the tendon increases, thus reducing the extensor lag. The extensor lag of the digit can be compensated by the motion of the wrist movement. Consequently, the adhesion can have a more direct influence on the single-digit-force than on the range of motion of the digit. This may explain why many of the patients’ injured fingers exhibited a minimal extensor lag (mean of 5°) yet had a small normalized single-digit-force ratio compared to their healthy contralateral fingers (mean of 0.74).

The AI of the healthy participants was observed to be lower than that of the patients. One patient exhibited an AI that was similar to the typical value found in the healthy participants, which was probably because of that patient having already finished his rehabilitation and visiting the hospital for a follow-up session. The clear difference in the AI of the healthy participants and that of the patients also suggests that it may be valuable information for clinicians to use in evaluating the completion patients’ rehabilitation process.

Decreased tendon gliding severely affects hand function and presents a challenge to clinicians. The primary goal of rehabilitation is the maintenance of maximal tendon gliding and range of motion. According to the rehabilitation protocol, active and passive mobilization are implemented during the 4–8 week period following surgery. When a suspected adhesion develops, it may be necessary for a patient to undergo resistance exercise to address the limited tendon gliding. Therefore, the ability of the proposed AI to identify the location of the suspected adhesion by observing the adhesion image is an essential development for clinicians. Furthermore, a potential use of the proposed AI is for the injection of antiinflammatories and lubricants to reduce postsurgical adhesion^34^. Clinicians remain highly reliant on manual observation for the identification of locations for injection; the proposed AI can provide clinicians with a more accurate means for identifying locations suspected of adhesion and thereby improve the effectiveness of the rehabilitation process. Moreover, by comparing the differences among the adhesion images obtained from each measurement, clinicians can easily visualize the progress of the rehabilitation. Thus, the ability of the proposed AI method to identify the specific location and total coverage of an adhesion enables clinicians to make better-informed decisions during rehabilitation and is thus a potentially useful diagnostic tool.

Several limitations of the present study should be addressed. First, the study sample was small (involving only eight cases over 1 year); this was partly because of the application of strict inclusion criteria. Nevertheless, the AI was found to be strongly correlated with the two additional clinical measurements used for evaluating the extent of adhesion; moreover, clear and statistically significant differences in the AI among healthy individuals, overall patients, and the first and second measurements of the patients were observed. Consequently, this limitation did not adversely affect the results obtained in this study. Second, the intra- and interrater reliabilities were not considered. However, because the EDC tendon is very close to the surface of the hand, it can be visually identified, making it relatively easy to locate compared to other tendons in the human body. Third, previous studies have found that joint positions, movement velocity, and single-digit motion influence the extent of relative displacement between the tendon and its surrounding tissue^21^,^35^,^36^,^37^. However, the preceding variables were not included in the present study for the following reasons: For joint motion, the intrinsic-plus and intrinsic-minus position were considered a safer and more stable position for patients with metacarpal fracture. In addition, because most patients involved in the present study exhibited limited hand movement, a relatively slow speed (5 mm/s) was employed to allow comfortable movement. Moreover, because the EDC tendon usually has tendinous and fascial connections between its tendons on the dorsal surface of the hand^24^, a multiple digit movement was used to produce a larger displacement of the tendon. Moreover, manipulating these variables may offer additional benefits. Additional experiments should be conducted. Fourth, patients with a wide range of injury durations were included. However, the strong correlations between AI and the two additional clinical measures (extensor lag and single-digit-force) indicate that AI can effectively assess the rehabilitation status of patients with a wide range of injury durations.

In conclusion, this study has demonstrated that the relative displacement between the EDC tendon and its surrounding tissue provides valuable information for evaluating the extent of adhesion and differentiating the locations of suspected adhesion formation in patients with metacarpal fractures. The proposed AI has potential for clinical application as a diagnostic tool for rehabilitation.

## Additional Information

**How to cite this article**: Lai, T.-Y. *et al.* A Novel Adhesion Index for Verifying the Extent of Adhesion for the Extensor Digitorum Communis in Patients with Metacarpal Fractures. *Sci. Rep.* **6**, 31102; doi: 10.1038/srep31102 (2016).

## Figures and Tables

**Table 1:** Parameter settings for the block matching method.

Reference template and Candidate window size (pixels)	ROI size (pixels)	Ultrasound data (mm/pixels)
6 × 16	26 × 56	0.0735

**Table 2:** Values of the AI, extensor lag, and single-digit-force for healthy individuals and for patients between the two measurements.

	Healthy individuals *N* = 39	Patients *N* = 8		*P*†
		First measurement	Second measurement	
AI (%)	19.6 ± 10.3	69.54 ± 12.06	55.41 ± 20.38	0.017*
Extensor lag (°)	—	18.13 ± 10.33	8.75 ± 8.35	0.011*
Single-digit-force (%)	—	0.59 ± 0.24	0.73 ± 0.32	0.025*

^†^Comparison between first and second measurement using the Wilcoxon signed-rank test.

*P < 0.05. Data are mean ± SD values.

**Table 3:** Demographic information of the patients and healthy individuals.

		Healthy individuals	Patients	*P*†
Age (years)		36 ± 14	39 ± 17	0.569
Sex	Male	20 (51.3)	6 (75.0)	0.269
	Female	19 (48.7)	2 (25.0)	
Hand involved	Right	36 (92.3)	6 (75.0)	0.196
	Left	3 (7.7)	2 (25.0)	
Digit involved	Index	2 (5.1)	1 (12.5)	0.219
	Ring	31 (79.5)	4 (50)	
	Little	6 (15.4)	3 (37.5)	
Injury duration	<3 months	–	3 (37.5)	–
	3–6 months	–	4 (50)	
	>6 months	–	1 (12.5)	

Data are mean ± SD or *N* (%) values. ^†^Comparison between parameters using the chi-squared test.
